# Deletion of Pregnancy Zone Protein and Murinoglobulin-1 Restricts the Pathogenesis of West Nile Virus Infection in Mice

**DOI:** 10.3389/fmicb.2019.00259

**Published:** 2019-02-13

**Authors:** Keeton Krause, Francine Azouz, Eileen Nakano, Vivek R. Nerurkar, Mukesh Kumar

**Affiliations:** ^1^Department of Tropical Medicine, Medical Microbiology and Pharmacology, Pacific Center for Emerging Infectious Diseases Research, John A. Burns School of Medicine, University of Hawai‘i at Mānoa, Honolulu, HI, United States; ^2^Department of Biology, College of Arts and Sciences, Georgia State University, Atlanta, GA, United States

**Keywords:** West Nile virus, flavivirus, alpha-macroglobulins, pregnancy zone protein, murinoglobulin-1, neuroinflammation, host–pathogen interaction, virus replication

## Abstract

West Nile virus (WNV) is an enveloped positive-stranded RNA virus that causes meningitis, encephalitis, and acute flaccid paralysis in humans. There are no therapeutic agents available for use against WNV infection. Alpha-2 macroglobulin (A2M) is a major plasma proteinase inhibitor that also has important role in immune modulation. In mice, pregnancy zone protein (PZP) and murinoglobulin-1 (MUG-1) are two close homologous of human A2M. In this study, we investigated the role of PZP and MUG-1 proteins in the pathogenesis of WNV infection in mice. Adult C57BL/6J wild-type and PZP/MUG-1 double knockout (DKO) mice were inoculated subcutaneously with WNV and mortality, virus burden, and immune responses were analyzed. Infection of wild-type (WT) mice with WNV resulted in significantly high morbidity and mortality. In comparison, no mortality was observed in DKO mice, suggesting that PZP and MUG-1 play a deleterious role in WNV infection. Increased survival in WNV-infected DKO mice was associated with significantly low viral burden in serum, spleen, kidney, and brain compared to WT mice. In addition, significantly reduced levels of type 1 interferon and WNV-specific antibodies were observed in the DKO mice compared to WT mice. We further demonstrated that protein levels of inflammatory cytokines and chemokines in the serum, spleen, and brain were significantly reduced in DKO mice compared to WT mice. Collectively our data demonstrate that lack of PZP and MUG-1 restricts the pathogenesis of WNV infection in mice.

## Introduction

West Nile virus (WNV) is a single-stranded positive-sense RNA virus that targets neurons to cause potentially lethal encephalitis. WNV is related to other important flavivirus pathogens such as dengue virus, Japanese encephalitis virus (JEV) and yellow fever virus. WNV is an enveloped virus with genome length of 11 kb encoding a large single polypeptide which is cleaved into envelope glycoprotein E, transmembrane protein M, capsid protein, and several non-structural proteins. Since its initial introduction into New York in 1999, outbreaks of WNV-associated encephalitis (WNVE) have occurred in all 48 contiguous states, resulting in hundreds of deaths (Brinton, 2002). The fatality rate is approximately 10% for hospitalized encephalitic cases with increased risk in patients with compromised immune systems, including AIDS and transplant patients, older age and having underlying conditions of diabetes mellitus (Hayes and Gubler, 2006). WNV continues to spread and cause human disease in new areas of the world. Currently, there is no specific treatment or vaccine exist for treating individuals with WNV infection, and the WNV disease pathogenesis is not completely understood.

The alpha-macroglobulins family in humans consists of alpha-1 macroglobulin (A1M), alpha-2 macroglobulin (A2M), pregnancy zone protein (PZP), and complement components (C3, C4, and C5) (Rehman et al., 2013). A2M and PZP are proteinase-inhibiting glycoproteins of human plasma. A2M is one of the most abundant protein in human plasma with a concentration of 2–4 mg/mL of plasma (Rehman et al., 2013). A2M acts as a binding and carrier protein for a large number of growth factors, cytokines, hormones, disease factors, and various small molecule nucleophilic ligands (Abe et al., 1989; Wu et al., 1998; Rehman et al., 2013). Dysregulation of A2M has been linked to numerous disorders such as inflammatory bowel disease, stroke, Hepatitis C virus associated-liver fibrosis, tissue inflammation, Alzheimer disease, and several cytokine-related diseases (Tiggelman et al., 1997; Wu et al., 1998; Kovacs, 2000; Rehman et al., 2013). Mouse A2M, mouse PZP, and murinoglobulin-1 (MUG-1) are the close homologous of human A2M in mice. In adult mice, PZP and MUG-1 proteins are found at high concentrations in the plasma. However, A2M protein is barely detectable in adult mice (Bernhard et al., 2007; Lee et al., 2012). Several studies have reported that whereas A2M expression in the liver of mice and rats is high during development, it is not detectable in adult animals (Kodelja et al., 1986; Fletcher et al., 1988; Lee et al., 2012). Like human A2M, mouse PZP is highly expressed in the liver of adult mice (Bernhard et al., 2007; Lee et al., 2012). Both human A2M and mouse PZP proteins are tetramers. Murine PZP also inhibits proteases from all known classes and undergoes similar human A2M conformational changes upon reaction with protease. MUG-1 is a single chain proteinase inhibitor, which is also found at high concentrations in adult murine plasma (1–2 mg/mL). MUG-1 is a monomeric form of human A2M. With nearly identical function to human A2M, MUG-1 is a murine-specific protein with no corresponding protein in human (Abe et al., 1989; Webb et al., 1996; Bernhard et al., 2007). Therefore, mouse PZP and MUG-1 proteins represent the role of A2M in human plasma.

Human A2M is known to bind and enhance internalization of different toxins and pathogens such as bacteria or virus (Liao et al., 2002). Viral proteins conjugated to A2M are taken up by antigen presenting cells more effectively than the free viral proteins (Mitsuda et al., 1995). It has been demonstrated that A2M binds to DENV virions of all four serotypes and enhances DENV-2 infectivity *in vitro* (Huerta et al., 2014). Similarly, it has been shown that A2M binds to HSV-1 particles and facilitates internalization of HSV resulting in increase in the synthesis of viral proteins in the neuronal cell line (Alonso et al., 2001). Moreover, HIV-1 envelope protein conjugated to A2M is effectively taken up by macrophages, which results in an increased production of specific antibodies against the peptide (Mitsuda et al., 1993; Liao et al., 2002).

Although A2M is known to bind and internalize viral proteins and modulate immune response, and has been demonstrated to enhance virus infectivity *in vitro*, its *in vivo* function in viral infection has yet to be defined. We have previously reported that WNV infection induced upregulation of alpha-macroglobulins in mice (Kumar et al., 2016). Murine PZP and MUG-1 represent the role of A2M in human plasma. To define the *in vivo* role of these proteins in WNV infection, we investigated the susceptibility of mice deficient in PZP and MUG-1 against WNV infection.

## Materials and Methods

### Animals

C57BL/6 J (WT) mice and PZP^-/-^/MUG1^-/-^ mice (DKO mice) on C57BL/6J background were purchased from The Jackson Laboratory (Bar Harbor, ME, United States). All animal experiments were conducted in the animal biosafety level-3 laboratory. This study was carried out in accordance with the regulations of the National Institutes of Health and the Institutional Animal Care and Use Committee (IACUC). The protocol was approved by the University of Hawaii IACUC (Protocol number 15-2202).

### WNV Infection Experiments and Plaque Assay

For survival studies, WT and DKO mice were inoculated subcutaneously via the footpad route with 1,000 or 100 plaque-forming units (PFU) of WNV as described previously (Kumar et al., 2013, 2014a). Clinical symptoms such as ruffled fur, hunchbacked posture, paralysis, tremors, and ataxic gait were observed twice a day. In independent experiments, mice were inoculated with PBS or 100 PFU of WNV, and at specific days, mice were anesthetized and perfused with PBS, and tissues were harvested. WNV titers were measured by plaque assay as described previously (Verma et al., 2009; Kumar et al., 2012).

### qRT-PCR

Virus titers were analyzed in the brain by qRT-PCR. qRT-PCR was conducted using primers and probes specific for the WNV envelope region as described previously (Roe et al., 2012; Kumar et al., 2013). For IBA1 gene expression analysis, cDNA was prepared using iScript^TM^ cDNA Synthesis Kit (Bio-Rad), and qRT-PCR was conducted as described previously (Kumar et al., 2013). Primer sequence used: Forward TGATTCTGATGTATGAGGAG, Reverse GGAGCGTCATTTATTTAGTC.

### WNV-Specific IgM and IgG Antibodies

Microsphere immunoassay (MIA) using WNV envelope E protein was used to quantify titers of WNV-specific antibodies as described previously (Namekar et al., 2012; Kumar et al., 2015).

### Interferon ELISA

Protein levels of IFN-α and IFN-β were measured using the VeriKine^TM^ Mouse Interferon-α ELISA Kit and VeriKine^TM^ Mouse Interferon-β ELISA Kit (PBL Interferon Source) as described previously (Kumar et al., 2012).

### Measurement of Cytokines and Chemokines

Protein levels of inflammatory cytokines and chemokines were measured using multiplex immunoassay kit (MILLIPLEX MAP Mouse Cytokine/Chemokine Kit, Millipore) (Kumar et al., 2012, 2014b; Kumar and Nerurkar, 2014).

### Statistical Analysis

GraphPad Prism 5.0 was used to perform a Kaplan Meier log-rank test to compare survival curves. Mann–Whitney test and unpaired Student’s *t*-test using GraphPad was used to calculate p values for clinical score, plaque assay, ELISA and multiplex immunoassay. Differences with *P*-values of <0.05 were considered significant.

## Results

### Deletion of PZP and MUG-1 Restricts the Pathogenesis of WNV Infection in Mice

**FIGURE 1:**
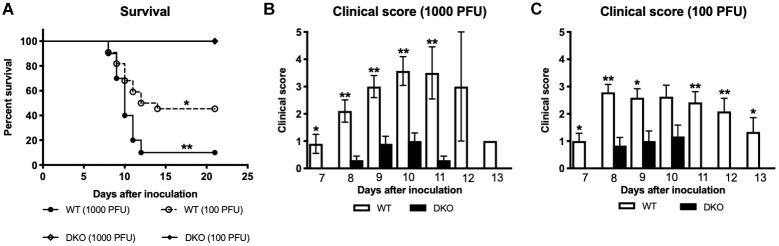
Survival analysis and clinical score of WT and DKO mice following West Nile virus (WNV) infection. **(A)** WT and DKO mice were inoculated subcutaneously with 1,000 or 100 PFU of WNV. The survival difference between WT and DKO mice was statistically significant (*n* = 12–22 mice per group). **(B,C)** Animals were monitored twice daily for clinical signs as described in materials and methods. Error bars represent SEM. ^∗^*p* < 0.05, ^∗∗^*p* < 0.001.

We inoculated C57BL/6J (WT) and mice deficient in PZP and MUG-1 (hereafter referred as; double knockout mice or DKO) with 1,000 or 100 PFU of WNV via subcutaneous route. Animals were monitored twice daily for morbidity and mortality. Infection of WT mice with 1,000 PFU of WNV NY99 was highly lethal and resulted in 90% mortality. Similarly, WT mice inoculated with 100 PFU of WNV NY99 also exhibited high (54%) mortality. In comparison, no mortality was observed in DKO mice infected with 1,000 or 100 PFU of WNV, indicating a detrimental role for PZP and MUG-1 proteins during WNV infection (Figure 1A). As depicted in Figure 1B,C, both WT and DKO mice demonstrated clinical signs of infection, however, severe neurological symptoms were observed only in WT mice. All surviving animals were positive for anti-WNV neutralizing antibodies.

### WNV Replication in WT and DKO Mice

**FIGURE 2:**
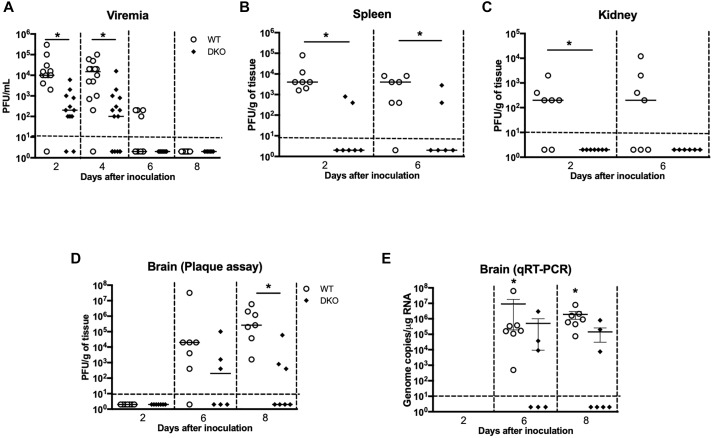
Viral burden in the serum, peripheral tissues, and brain of WT and DKO mice. Viral loads were measured in the **(A)** serum, **(B)** spleen, **(C)** kidney, and **(D)** brain and expressed as PFU/mL of serum and PFU/g of tissue. Data points below the horizontal dotted line are negative (*n* = 12–22 mice per group). ^∗^*p* < 0.05. **(E)** WNV copy number in the brain was determined by qRT-PCR. The data are expressed as genome copies/μg of RNA ± SEM (*n* = 6–7 mice per group). ^∗^*p* < 0.05.

To better understand how PZP and MUG-1 mediate WNV pathogenesis, we measured WNV load in the serum, spleen, kidney, and brain of the WT and DKO mice at various time points after WNV inoculation. Consistent with the survival result, WNV titers in the serum were significantly lower in DKO mice when compared to WT mice (Figure 2A). Virus titers assayed in the spleen also followed a similar trend, and DKO mice spleens had significantly lower virus titers than WT mice (Figure 2B). Virus replication kinetics observed in the kidneys of WT and DKO mice was similar to the spleen (Figure 2C). We next measured virus titers in the brains. WNV was undetectable in the brains of both WT and DKO mice at day 2 (Figure 2D). High viral load was observed in the brains of 86% of the WT mice at day 6 by plaque assay. In comparison, only 50% of the DKO mice had measurable virus titer at day 6. Similarly, at day 8, significantly high virus titer was observed in all of the WT mice. However, low levels of virus were present in only 50% of the DKO mice at day 8. WNV titer was significantly higher in WT mice than in DKO mice at day 8 after inoculation (Figure 2D). We also measured WNV RNA copies in the brains of WT and DKO mice. Similar to plaque assay, WNV RNA was significantly higher in WT mice than in DKO mice (Figure 2E).

### Antiviral Immune Responses in WT and DKO Mice

**FIGURE 3:**
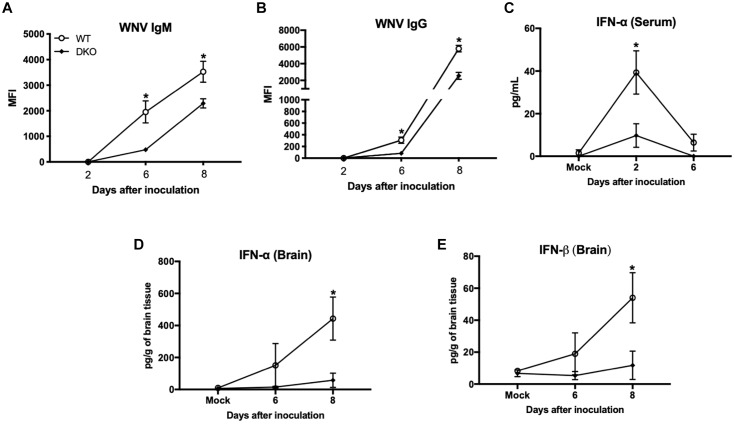
West Nile virus-specific immune responses in WT and DKO mice. Titers of IgM **(A)** and IgG **(B)** antibodies were measured by MIA using WNV E antigen. Data are expressed as median fluorescent intensity (MFI) ± SEM. **(C)** IFN-α levels were measured in the serum using mouse IFN-α ELISA kit. Data represents the mean concentration (pg/mL) ± SEM. **(D)** IFN-α, and **(E)** IFN-β levels were measured in the brain homogenates using mouse IFN-α and IFN-β ELISA kits. Data represents the mean concentration (pg/g of tissue) ± SEM (*n* = 6–7 mice per group). ^∗^*p* < 0.05.

We next measured titers of IgM and IgG antibodies in the serum using MIA. Consistent with previous studies, anti-WNV IgM and IgG were first detected at day 6 and gradually increased at day 8. Titers of both IgM and IgG antibodies were significantly higher in WT mice compared to DKO mice (Figure 3A,B).

We next measured the levels of type 1 IFN in the serum and brain homogenates of WT and DKO mice. High levels of IFN-α were detected in the serum of WT mice at day 2, which then decreased at day 6 after inoculation. In contrast, DKO mice did not elicit strong IFN response at day 2 after inoculation, which was significantly low in comparison to WT mice (Figure 3C). In the brain, WT mice developed high interferon response at day 6 after inoculation, which increased further at day 8 after inoculation. Similar to serum, DKO mice did not elicit a strong IFN response in the brain and levels of IFN-α and IFN-β were significantly low in comparison to WT mice at day 8 after inoculation (Figure 3D,E).

### Peripheral Cytokine Responses Are Blunted in DKO Mice

**FIGURE 4:**
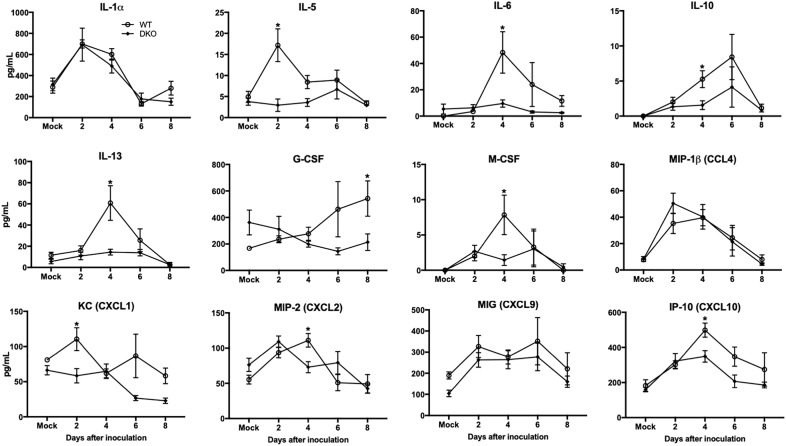
Cytokines and chemokines levels in the serum of WT and DKO mice. Levels of cytokines and chemokines as noted in the figure were measured in serum. Data represents the mean concentration (pg/mL) ± SEM (*n* = 7–16 mice per group). ^∗^*p* < 0.05.

Increased production of cytokines is a major pathological event associated with WNV infection in mice (Shrestha et al., 2008; Kumar et al., 2010; Suthar et al., 2013). Consequently, we examined the protein levels of key pro-inflammatory cytokines and chemokines in the serum and spleen of WT and DKO mice. WNV infection resulted in a dramatic induction of multiple cytokines and chemokines in the serum of WT mice at days 2 and 4 (Figure 4). However, unlike WT mice, infected DKO mice produced significantly lower levels of cytokines and chemokines. The levels of interleukin (IL)-6, -10, -13, and M-CSF were significantly higher in WT mice than DKO mice at day 4 after inoculation (Figure 4). Similarly, the levels of CXCL2 and CXCL10 were significantly higher in WT mice than DKO mice at day 4 after inoculation. However, no difference was observed in the levels of IL-1α, CCL4, and CXCL9 between both groups (Figure 4).

**FIGURE 5:**
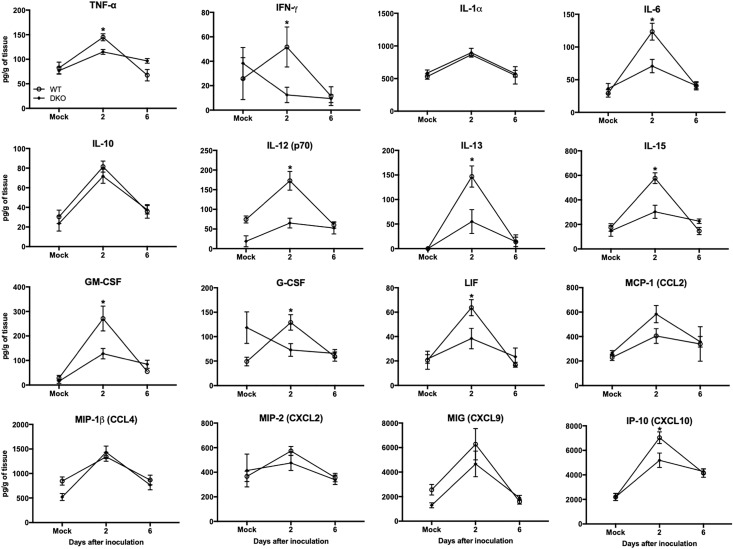
Cytokines and chemokines levels in the spleens of WT and DKO mice. Levels of cytokines and chemokines as noted in the figure were measured in spleen homogenates. Data represents the mean concentration (pg/g of tissue) ± SEM (*n* = 6–7 mice per group). ^∗^*p* < 0.05.

In spleen, WNV infection resulted in increased production of multiple cytokines and chemokines in WT mice at day 2 (Figure 5). However, unlike WT mice, cytokine responses were blunted in DKO mice. The levels of TNF-α, IFNγ, IL-6, IL-12, IL-13, IL-15, GM-CSF, LIF, CXCL10, and G-CSF were significantly higher in WT mice than DKO mice. No significant difference was observed in the levels of IL-1α, IL-10, CCL4, CXCL2, and CXCL9 between WT mice and DKO mice. These results correlate with reduced virus replication observed in the periphery of DKO mice compared to WT mice.

### DKO Mice Exhibit Reduced Inflammation in the CNS

**FIGURE 6:**
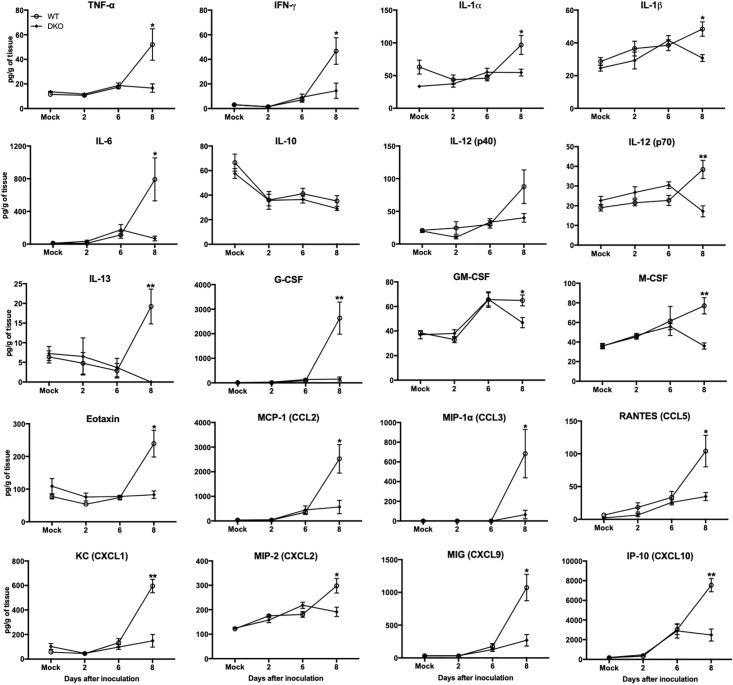
Cytokines and chemokines levels in the brains of WT and DKO mice. Levels of cytokines and chemokines as noted in the figure were measured in brain homogenates. Data represents the mean concentration (pg/g of tissue) ± SEM (*n* = 6–7 mice per group). ^∗^p < 0.05, ^∗∗^*p* < 0.001.

**FIGURE 7:**
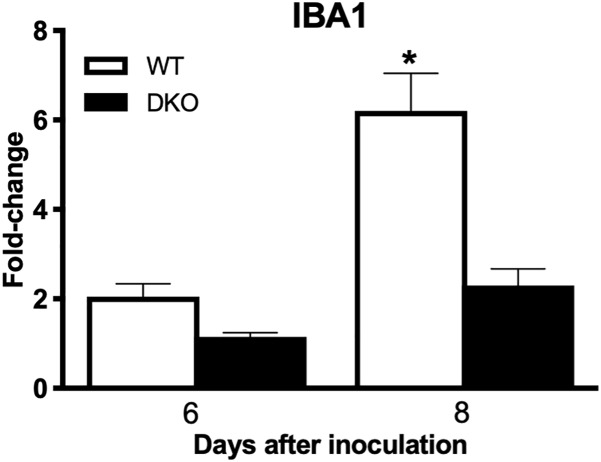
IBA1 expression levels in the brains of WT and DKO mice. qRT-PCR was conducted to determine the fold change in IBA1 gene expression. Change in the levels of IBA1 was first normalized to the β-actin gene and then the fold-change in infected brains was calculated in comparison to corresponding mock-infected brains. Data represents the mean ± SEM, representing two independent experiments. ^∗^*p* < 0.05.

In brain, WNV-induced production of cytokines and chemokines was not observed in the DKO mice (Figure 6). The levels of cytokines such as TNF-α, IFNγ, IL-1α, IL-1β, IL-6, IL-12 (p70), IL-13, G-CSF, and M-CSF were significantly lower in the DKO mice than WT mice at day 8 after inoculation. In addition, the levels of chemokines such as eotaxin, CCL2, CCL5, CXCL1, CXCL2, CXCL9, and CXCL10 were significantly reduced in the DKO mice than WT mice. However, the levels of IL-10 and IL-12 (p40) did not differ between both the groups (Figure 6). To see the correlation between enhanced pro-inflammatory mediators and microglial cells number in the brain, we conducted qRT-PCR to measure IBA1 transcript levels. Our data demonstrate that mRNA expression of IBA1 is significantly higher in WT mice compared to DKO at day 8 after inoculation (Figure 7).

## Discussion

In this study, we demonstrate that lack of PZP and MUG-1 proteins restricts the pathogenesis of WNV infection in mice. All PZP^-/-^/MUG1^-/-^ mice (DKO mice) survived subcutaneous WNV infection, and demonstrated reduced viral burden in serum, spleen, kidney, and brain. These observations were associated with significantly attenuated antibody, interferon, and inflammatory responses in the periphery and the brain of DKO mice.

In adult mice, PZP and MUG-1 (Abe et al., 1989; Bernhard et al., 2007; Lee et al., 2012; Rehman et al., 2013) are two close homologous of human A2M. Therefore, in this study we used mice deficient in both PZP and MUG-1. This mouse model has been previously used to understand the role of mouse PZP and MUG-1 proteins in acute pancreatitis, *Trypanosoma cruzi* infection, and drug pharmacokinetics (Umans et al., 1999; Waghabi et al., 2002; Shemesh et al., 2016). In our study, PZP and MUG-1 knockout mice exhibited significantly lower levels of virus titers in the serum, peripheral organs and brain. This is in agreement with previous observations demonstrating that PZP and MUG-1 deficient mice had significantly lower parasitemia than WT mice after infection with *T. cruzi* (Waghabi et al., 2002). Waghabi et al. (2002) reported similar results using single knockout of mouse PZP. In addition, mice deficient in mouse PZP alone are also resistant to lethal infection by *Klebsiella pneumoniae* and had significantly less bacteria in the blood and in different organs than in WT mice (Hochepied et al., 2002).

It is well documented that WNV-induced pro-inflammatory cytokines and chemokines play critical roles such as infiltration of immune cells into the brain, activation of glial cells, and neuronal death (Shrestha et al., 2008; Kumar et al., 2010; Suthar et al., 2013). In this study, we observed reduced levels of inflammatory mediators such as IL-1β, IL-6, TNF, IL-1α, IFN-γ, CCL5, CXCL10 in the serum, spleen and brain of DKO mice compared to WT mice after WNV infection. These results indicate that reduced virus replication in DKO mice resulted in lower inflammatory response in the periphery and brain. Since, A2M has been used as an adjuvant to enhance immune response, we also measured levels of WNV-specific antibodies and type 1 IFN in the WT and DKO mice. Our data demonstrate that titers of WNV-specific IgM and IgG antibodies were significantly lower in DKO mice. Moreover, DKO mice did not elicit strong interferon response and levels of IFN-α and IFN-β were significantly lower in comparison to WT mice. These results correlate with reduced virus replication observed in the periphery and brain of DKO mice compared to WT mice.

Viral proteins conjugated to A2M are taken up by antigen presenting cells more effectively than the free viral proteins (Liao et al., 2002). A2M has been shown to interact with the envelope protein of dengue virus and enhances virus infectivity *in vitro* (Huerta et al., 2014). Similarly, it has been shown that A2M binds to HSV-1 particles and facilitates internalization of HSV resulting in increase in the synthesis of viral proteins in the neuronal cell line (Alonso et al., 2001). A2M binds to its specific receptor on the cell surface of many cell types (Kounnas et al., 1992; Rehman et al., 2013). It is possible that A2M facilitates internalization of WNV through the low-density lipoprotein (LDL) receptor related protein (LRP), a heterodimer glycoprotein and receptor of A2M (Kounnas et al., 1992). HCV and other members of Flaviviridae are endocytosed via the LDL receptor (Agnello et al., 1999). It has been demonstrated that binding of HIV-1 transactivator (Tat) protein to LRP promotes efficient uptake of Tat into neurons (Liu et al., 2000). Future studies are warranted to delineate the potential role of A2M as a shuttle for WNV to LRP as a receptor complex that may mediate virus endocytosis.

## Conclusion

In conclusion, our data demonstrate the critical role of PZP and MUG-1 proteins during WNV infection in mice. Based on our data, we conclude that reduced virus replication in DKO mice resulted in lower inflammatory response, thereby leading to increased survival with less severe clinical symptoms in these mice compared to WT mice. Based on our study, we cannot determine the individual role of PZP and MUG-1 in WNV pathogenesis. One major limitation is the further need for mechanistic studies to understand how PZP and MUG-1 independently modulate the WNV infection. Therefore, additional studies, including binding of alpha-macroglobulin proteins with WNV and effect on the virus entry and replication, are warranted to understand the underlying mechanism.

## Author Contributions

MK and VN designed the experiments, analyzed the data, and wrote the manuscript. KK, FA, EN, and MK conducted the experiments. KK, FA, VN, and MK analyzed the data. All authors have read and approved the final version of the manuscript.

## Conflict of Interest Statement

The authors declare that the research was conducted in the absence of any commercial or financial relationships that could be construed as a potential conflict of interest.
